# Multimorbidity and use of hypnotic and anxiolytic drugs: cross-sectional and follow-up study in primary healthcare in Iceland

**DOI:** 10.1186/s12875-016-0469-0

**Published:** 2016-06-06

**Authors:** Kristjan Linnet, Larus S. Gudmundsson, Frida G. Birgisdottir, Emil L. Sigurdsson, Magnus Johannsson, Margret O. Tomasdottir, Johann A. Sigurdsson

**Affiliations:** Centre of Development, Primary Health Care of the Capital Area, Reykjavik, Iceland; Clinical Quality and Services, Directorate of Health, Reykjavik, Iceland; Faculty of Pharmaceutical Sciences, School of Health Sciences, University of Iceland, Reykjavik, Iceland; Centre of Public Health Sciences, School of Health Sciences, University of Iceland, Reykjavik, Iceland; Department of Family Medicine, School of Health Sciences, University of Iceland, Reykjavik, Iceland; Department of Pharmacology and Toxicology, Faculty of Medicine, School of Health Sciences, University of Iceland, Reykjavik, Iceland; Faculty of Medicine, Department of Public Health and General Practice Research Unit, Norwegian University of Science and Technology, Trondheim, Norway

**Keywords:** Hypnotics, Anxiolytics, Multimorbidity, ICD-10, Primary care, Chronic health problems, Multiple chronic conditions, Insomnia

## Abstract

**Background:**

The prevalence of multimorbidity is increasing worldwide, presumably leading to an increased use of medicines. During the last decades the use of hypnotic and anxiolytic benzodiazepine derivatives and related drugs has increased dramatically. These drugs are frequently prescribed for people with a sleep disorder often merely designated as “insomnia” in the medical records and lacking a clear connection with the roots of the patients’ problems. Our aim was to analyse the prevalence of multimorbidity in primary healthcare in Iceland, while concurrently investigating a possible association with the prevalence and incidence of hypnotic/anxiolytic prescriptions, short-term versus chronic use.

**Methods:**

Data were retrieved from a comprehensive database of medical records from primary healthcare in Iceland to find multimorbid patients and prescriptions for hypnotics and anxiolytics, linking diagnoses (ICD-10) and prescriptions (2009–2012) to examine a possible association. Nearly 222,000 patients, 83 % being local residents in the capital area, who contacted 16 healthcare centres served in total by 140 general practitioners, were set as a reference to find the prevalence of multimorbidity as well as the prevalence and incidence of hypnotic/anxiolytic prescriptions.

**Results:**

The prevalence of multimorbidity in the primary care population was 35 %, lowest in the young, increasing with age up to the 80+ group where it dropped somewhat. The prevalence of hypnotic/anxiolytic prescriptions was 13.9 %. The incidence rate was 19.4 per 1000 persons per year in 2011, and 85 % of the patients prescribed hypnotics/anxiolytics were multimorbid. Compared to patients without multimorbidity, multimorbid patients were far more likely to be prescribed a hypnotic and/or an anxiolytic, OR = 14.9 (95 % CI = 14.4–15.4).

**Conclusions:**

Patients with multiple chronic conditions are common in the primary care setting, and prevalence and incidence of hypnotic/anxiolytic prescriptions are high. Solely explaining use of these drugs by linear thinking, i.e. that “insomnia” leads to their prescription is probably simplistic, since the majority of patients prescribed these drugs are multimorbid having several chronic conditions which could lead to sleeping problems. However, multimorbidity as such is not an indication for hypnotics, and doctors should be urged to greater caution in their prescribing, bearing in mind non-pharmacological therapy options.

## Background

In recent years the extensive research on the epidemiology of multimorbidity in several countries has revealed that it is an extremely common problem, not least in primary healthcare where the majority of patients have more than one problem at any given time [1–3]. The percentage of patients with multiple diagnoses increases as people get older. However, the extent of multimorbidity in young people and even in children is perhaps greater than might be expected, probably because risk factors for multiple diseases are not randomly distributed [1, 4]. Multimorbidity can be measured in several different ways and the range of diseases included varies between studies. A recent study with health data classified according to ICPC-2 chapters (International Classification of Primary Care, Version 2), ICD-10 chapters (International Classification of Diseases, 10th edition) or CIRS domains (Cumulative Illness Rating Scale) found that when multimorbidity is defined as two or more disease entities, prevalence estimates are similar, no matter what diagnostic classification system is used [5].

Multimorbidity has been associated with polypharmacy [6] and lower socioeconomic status [7]. Studies have shown that clustering of diseases or conditions in persons with multimorbidity is complex and does not follow any typology [8–10]. This could indicate a common root of chronic biological dysregulation in the body, better described as allostatic overload, i.e. a physiological scenario where the organism’s adaptive and restorative capacity is overtaxed [8, 10].

A number of diseases are correlated with insomnia and thus increasing multimorbidity might lead to an increase in sleep disorders. Hypnotic and anxiolytic benzodiazepine derivatives are commonly prescribed to people with a sleep disorder often just labelled with the diagnosis “insomnia” in the medical records. However, in epidemiological studies on prescribing habits it is not always obvious what type of health problems lie behind or are possibly connected with prescribing hypnotic and anxiolytic drugs. This can be of great importance in the primary care setting as a holistic view of a patient’s health problems is usually put in focus.

Since the launching of chlordiazepoxide in 1960 and diazepam a few years later the number of benzodiazepine derivatives and related drugs released on the market has multiplied and their use both as anxiolytics and hypnotics increased dramatically [11]. Now they are some of the most widely prescribed drugs. This has caused much controversy with widely divided opinions, e.g. concerning adverse effects, dependence, abuse liability, efficacy and tolerability [11]. The serious side effects of benzodiazepines have been known for decades [12], and in recent years, concerns have been raised about possible links between the use of these drugs and increased mortality [13–16]. Nevertheless, an incontestable evidence of a causal relationship has not yet been presented, and findings in other studies have not lent support to a strong association of hypnotic use with excess mortality [17, 18]. A recently published prospective study found that the risk of mortality was not significantly associated with hypnotic use regardless of type and duration [19] while a retrospective cohort study which also corrected for a range of potential confounders found an association with significantly increased risk of mortality [20]. In a newly published study connecting disease burden and use of hypnotics, it was shown that there was a 50 % increase in the use of benzodiazepines 24 months before death. It was concluded that the association between use of these drugs and mortality is at least partially due to confounding [21]. Apart from the well-known risk of falls and fractures, there are other untoward effects such as a possible association with increased suicide risk [15, 22], increased risk of road traffic accidents [23], dementia [24] and Alzheimer’s disease [25]. Besides, the effectiveness of the newer type of hypnotics, the so-called Z drugs (zolpidem, zopiclone) has been questioned [26]. Thus, there are numerous risks associated with the use of these drugs which to some degree might be avoided by using other treatment options for insomnia instead of prescribing hypnotics. Sleep hygiene recommendations could be applied as first-line therapy [27] followed by interventions needing more specialised skills such as cognitive behavioural therapy [28].

The question might be raised whether patients with a heavy disease burden are more prone to becoming chronic users as sleeping problems leading to hypnotics being prescribed, may be a direct consequence of multimorbidity, although multimorbidity as such is not an indication for the prescription of these drugs. In a paper on the medicalisation of sleeplessness it was shown that during the study period insomnia diagnoses increased more than sevenfold while prescriptions for non-benzodiazepine sedative hypnotics (Z-drugs and ramelteon) increased about thirtyfold [29]. It is known that the use of benzodiazepine derivatives and related drugs increases with age [11] and also that the prevalence of multimorbidity increases significantly with age both in men and women [5–7]. It is also worth noting that in a study comparing measures of multimorbidity to predict outcomes in primary care, the number of drugs prescribed was a powerful predictor of mortality [30]. There have not been many studies on the relationship between selected drug classes and multimorbidity, but it has been shown that comorbidity was a correlate of benzodiazepine use in patients with depressive and/or anxiety disorders [31]. Although several epidemiological studies on multimorbidity have been done, there are still many countries, including Iceland until now, where little is still known about the prevalence of multimorbidity.

The aim of this study was firstly to analyse the prevalence of multimorbidity in primary healthcare, and secondly to analyse the prevalence and incidence of prescriptions of hypnotics and anxiolytics, mostly benzodiazepine derivatives and Z-drugs, in order to find a possible association between the use of these drugs and the prevalence of multimorbidity, based on the assumption that sleeping problems and disease discomfort related to multimorbidity might lead to prescription of these drugs although a clear-cut indication might be lacking. Thirdly, our aim was to find if incident patients took the drugs according to clinical guidelines (2–4 weeks) or if they had become either intermittent or regular users, thus indicating the possibility that multimorbidity might be a contributing factor in the chronic use of these drugs.

## Methods

### Design and setting

The Primary Health Care of the Capital Area (PHCCA) includes most of the primary healthcare centres in Reykjavik and nearby municipalities with a population of just over 200,000 serving general practice, maternity and well-child care, and is staffed by general practitioners (GPs), midwives, nurses and other personnel. The PHCCA operates a primary healthcare database of medical records from 16 healthcare centres including both prescriptions and diagnoses (ICD-10). Our study population, of whom 83 % had a postal code within the capital area, were all patients who contacted in total 140 GPs at these healthcare centres during a period of 4 years. All contacts with the primary healthcare centres were included in the study covering, in our judgement, the vast majority of primary care patients with multimorbidity and similarly primary care patients who were prescribed hypnotics/anxiolytics in the total population of the capital area.

A unique personal identifier (ID) is allocated to every inhabitant and this personal ID number is always entered when any information about a patient is recorded in the medical records database making it possible to link all stored information about the patient through the ID, and in our study making it possible to uniquely link diagnoses with prescriptions issued for a particular patient.

In this paper multimorbidity is defined as two or more concurrent chronic diseases within one patient [1–3]. Selected ICD-10 diagnoses for chronic conditions [8] as shown in Table 1, and data on the age and sex were extracted for all patients who consulted their family physician at any primary healthcare centre during the period 1 Jan 2009 to 31 Dec 2012 in order to find patients with multimorbidity.

**Table 1:** Chronic medical diseases/conditions according to ICD-10 considered relevant to multimorbidity in this study

Disease	ICD-10 code
Tuberculosis	A15–A19
Herpes zoster	B02
Human immunodeficiency virus	B20–B24
Cancer	C00–C97
Thyroidal diseases	E00–E07
Diabetes	E10–E14
Metabolic diseases	E65–E68
Hyperlipidaemia	E78
Mental health problems	F00–F99
Epilepsy	G40
Cardiovascular disease	I00–I09, I16–I99
Hypertension	I10–I15
Chronic obstructive pulmonary disease	J44
Asthma	J45–J46
Bronchiestasis	J47
Gastro-oesophageal reflux	K21
Psoriasis	L40
Rheumatoid arthritis	M05–M14
Osteoarthritis	M15–M19
Ankylosing spondylitis	M45
Chronic back pain	M53–M54
Fibromyalgia/myalgia	M79
Osteoporosis	M80–M82
Other chronic musculoskeletal problems	M00–M03, M20–M43, M46–M51, M60–M77, M83–M99
Renal disease	N18–N19

Prescriptions for hypnotics and anxiolytics from 1 Jan 2009 to 31 Dec 2012 were extracted from the medical records in order to find both their prevalence and incidence in the primary care patients, and to find the distribution according to age and sex. We defined incident patients as new users who had neither been prescribed a hypnotic nor an anxiolytic the previous 24 months [32]. Thus, a four-year period was needed for the purpose of calculating the incidence. We estimated drug consumption to be either in agreement with clinical guidelines (2–4 weeks) if there was only one prescription, intermittent if they were 2–4, or regular if the prescriptions were five or more during a 12 month period. Diagnoses for these patients were found by linking the information on both the prescriptions and the diagnoses in the medical records through the patient ID. Thus it was possible to find multimorbid patients, patients with one chronic condition and those who had no chronic diagnosis in this group of patients.

After having extracted these data the remaining patients in the primary healthcare database cohort had neither any chronic condition pertaining to the multimorbidity definition nor had they been prescribed any hypnotics/anxiolytics. We used all patients who contacted the healthcare centres during the same period (1 Jan 2009 to 31 Dec 2012) as a reference group when the prevalence was calculated for the multimorbidity and the hypnotics/anxiolytics prescriptions, respectively. Data on the mean age of the total population in Iceland was drawn from Statistics Iceland and compared with the PHCCA cohort [33].

### Collection of data on prescribed medications

Data on all medications classified in the Anatomical Therapeutic Chemical (ATC) classification system [34] as either anxiolytics (ATC classification N05B) or hypnotics and sedatives (ATC classification N05C) prescribed by general practitioners for their patients were extracted from the primary healthcare database as described in a previous study [35]. After collecting the data they were encrypted so the personal identity of the patients would not be revealed during the processing of the data set.

### Data analysis

The prevalence of the use of hypnotics/anxiolytics, mostly benzodiazepine and benzodiazepine-related drugs (Z-drugs) in the general population consulting the primary healthcare centres in the capital area was determined throughout the period from 1 Jan 2009 to 31 Dec 2012. The incidence was determined for patients initiating therapy in 2011 according to the above mentioned definition. The incidence is presented as number of cases per 1000 persons per twelvemonth period. Both the prevalence and the incidence were calculated according to age and sex. The number of patients prescribed hypnotics/anxiolytics was counted as well as the number of patients in this cohort who had multimorbidity.

To address a part of the clinical picture among multimorbid patients who initiated use of hypnotics/anxiolytics in 2011, and sorting them by diagnoses, we pooled together the diagnoses from Table 1 into three groups, i.e. mental disorders (ICD-10: F00-F99), pain related diagnoses (ICD-10: M00-M03, M05-M43, M45-M51, M53-M54, M60-M77, M79-M99 and N18-N19) and the rest of the diagnoses in Table 1 which we placed in a group called other diagnoses.

We used logistic regression to calculate age and sex specific odds ratios (OR) and 95 % confidence intervals (CI) for the likelihood of incident hypnotic/anxiolytic drug use, stratified by age and sex, during the period from 1 Jan 2009 to 31 Dec 2012, comparing those with and without multimorbidity during a 12 month period. Statistical analyses were conducted with Stata Release 13 (College Station, TX: StataCorp LP). Alpha level for statistical significance was set at 0.05.

## Results

A total of 221,822 patients contacted the primary healthcare centres during the period 1 Jan 2009 to 31 Dec 2012 or approximately two-thirds of the Icelandic population, 83 % being local residents. Table 2 shows the subset of patients with multimorbidity compared with the total cohort. Approximately 47 % of the total population did not have any relevant chronic condition as defined in Table 1, nearly 18 % had one chronic condition and 35 % were considered as having multimorbidity. The prevalence increased with age in both sexes up to 79 years of age.

**Table 2:** Number of patients and prevalence of multimorbidity

Age groups	All patients contacting the PHCCA			Patients with multimorbidity (number and %)					
	All	Men	Women	All	%	Men	%	Women	%
<1–19	61,033	30,982	30,051	6,157	10	3,156	10	3,001	10
20–29	34,255	15,950	18,305	8,250	24	3,174	20	5,076	28
30–39	33,291	16,310	16,981	11,077	33	4,480	27	6,597	39
40–49	27,619	13,498	14,121	12,934	47	5,406	40	7,528	53
50–59	25,300	12,398	12,902	14,657	58	6,456	52	8,201	64
60–69	19,159	9,351	9,808	11,955	62	5,578	60	6,377	65
70–79	11,166	5,197	5,969	7,637	68	3,440	66	4,197	70
80+	9,999	4,103	5,896	5,493	55	2,275	55	3,218	55
Total	221,822	107,789	114,033	78,160	35	33,965	32	44,195	39

All patients contacting the Primary Health Care of the Capital Area (PHCCA) during a period of 4 years (1 Jan 2009 to 31 Dec 2012) and prevalence of multimorbidity according to age and sex

During this four-year period a total of 30,926 patients were prescribed hypnotics/anxiolytics (prevalence: 13.9 %; 10.1 % for men and 17.5 % for women). The prevalence of hypnotics/anxiolytics prescriptions stratified for age and sex is shown in Table 3. It increases steadily with increasing age in both sexes until the highest age group is reached where it drops slightly.

**Table 3:** Prevalence of hypnotics/anxiolytics prescriptions

Age groups	Patients prescribed hypnotics/anxiolytics			Prevalence of hypnotics/anxiolytics use (%)		
	All	Men	Women	All	Men	Women
<1–19	848	389	459	1.4	1.3	1.5
20–29	2,748	994	1,754	8.0	6.2	9.6
30–39	4,071	1,480	2,591	12.2	9.0	15.3
40–49	4,881	1,626	3,255	17.7	12.0	23.0
50–59	6,154	2,094	4,060	24.3	16.9	31.5
60–69	5,382	1,938	3,444	28.1	20.7	35.1
70–79	3,948	1,388	2,560	35.4	26.7	42.9
80+	2,894	1,013	1,881	28.9	24.7	31.9
Total	30,926	10,922	20,004	13.9	10.1	17.5

Prevalence of hypnotics/anxiolytics prescriptions in primary care stratified by age and sex during the period 1 Jan 2009 to 31 Dec 2012. The total number of patients is shown in Table 2

In 2011 there were 4305 patients who had not been prescribed hypnotics/anxiolytics the two previous years who initiated treatment for insomnia or anxiety with a new prescription, hence categorised as incident patients. During a 12 month period approximately 56 % received one prescription only, 33 % received 2–4 prescriptions, and nearly 11 % of the patients were issued 5 prescriptions or more.

**Fig. 1:**
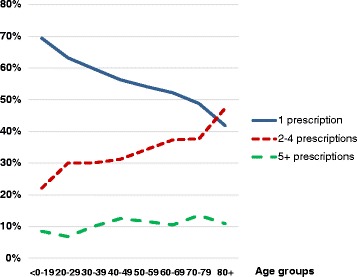
Proportion of incident patients issued one, 2–4 or 5+ prescriptions. The proportion of incident patients in the primary healthcare stratified by age who were prescribed hypnotics/anxiolytics for the first time in 2011 (no prior prescriptions for 24 months) grouped according to number of prescriptions during a 12 month period

Results for the incidence of hypnotics/anxiolytics prescriptions per 1000 persons per year in 2011 according to age and sex are shown in Table 4. The incidence increased with age and was higher in women than in men. Table 5 shows the division into 1 prescription, 2–4 prescriptions and 5+ prescriptions categories, both for all incident patients and the subset of incident multimorbid patients. The proportion of incident patients who received either one, 2–4 or 5+ prescriptions respectively during the 12 month period are plotted in Fig. 1.

**Table 4:** Incidence of hypnotics/anxiolytics prescriptions

Age	Men			Women			All
groups	N	Cases	pr 1,000	N	Cases	pr 1,000	pr 1,000
<1–19	30,982	107	3.5	30,051	128	4.3	3.9
20–29	15,950	215	13.5	18,305	375	20.5	17.2
30–39	16,310	268	16.4	16,981	542	31.9	24.3
40–49	13,498	258	19.1	14,121	485	34.3	26.9
50–59	12,398	287	23.1	12,902	509	39.5	31.5
60–69	9,351	231	24.7	9,808	340	34.7	29.8
70–79	5,197	139	26.7	5,969	201	33.7	30.4
80+	4,103	102	24.9	5,896	118	20.0	22.0
Total	107,789	1,607	14.9	114,033	2,698	23.7	19.4

Incidence of hypnotics and/or anxiolytics prescriptions in men and women in 2011 according to age and sex. No prior use for 24 months

**Table 5:** Number of hypnotics/anxiolytics prescriptions in different age groups

Age	All incident patients issued 1, 2–4 or 5+ prescriptions				Multimorbid incident patients issued 1, 2–4 or 5+ prescriptions			
groups	1	2–4	5+	All	1	2–4	5+	All
<1–19	163	52	20	235	82	28	7	117
20–29	373	177	40	590	225	124	29	378
30–39	484	244	82	810	348	188	62	598
40–49	418	232	93	743	335	187	78	600
50–59	431	273	92	796	362	243	81	686
60–69	298	213	60	571	262	198	54	514
70–79	166	128	46	340	151	119	44	314
80+	92	104	24	220	86	101	23	210
Total	2,425	1,423	457	4,305	1,851	1,188	378	3,417

Number of hypnotics and/or anxiolytics prescriptions in 2011 both in the total population of incident patients and in multimorbid incident patients solely

**Fig. 2:**
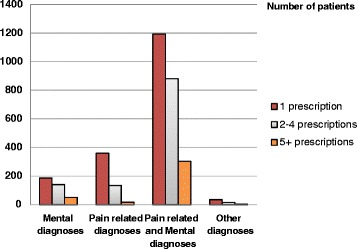
Diagnostic patterns among multimorbid incident patients. Distribution of mental diagnoses, pain related diagnoses and other diagnoses in multimorbid patients who initiated use of hypnotics/anxiolytics in 2011, orderd by diagnoses and either one time use (1), intermittent use (2–4) or regular use (5+)

Figure 2 illustrates some aspects of the clinical profile among the multimorbid incident patients subdivided into those who are either one time, intermittent or regular users. As shown, the combination of mental disorders and pain related diagnoses dominate the patient profile. Most often the use of these drugs was sporadic.

**Fig. 3:**
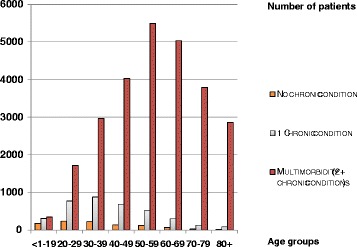
Number of patients prescribed hypnotics and/or anxiolytics. Patients prescribed hypnotics/anxiolytics according to age and number of chronic conditions

**Fig. 4:**
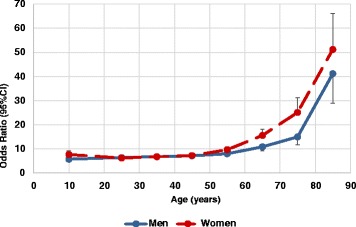
Odds ratios for being prescribed hypnotics and/or anxiolytics. The ORs with 95 % CIs for the likelihood of a hypnotic and/or an anxiolytic being prescribed for patients with multimorbidity according to age and sex

In the group of patients who were prescribed hypnotics/anxiolytics (30,926), 3 % (1018) had no chronic condition, 12 % (3693) had only one chronic condition while 85 % (26,215) were categorised as being multimorbid. Their distribution according to age, sex and number of conditions can be seen in Table 6 with the ORs for the likelihood of being prescribed a hypnotic and/or an anxiolytic if multimorbid. Figure 3 shows this group according to age and number of conditions and Fig. 4 shows the ORs by age and sex.

**Table 6:** Level of morbidity in patients prescribed hypnotics/anxiolytics according to age and sex

Age groups	Patients with no chronic disease			Patients with one chronic disease			Multimorbid patients – two or more chronic diseases				ORs (with 95 % CIs) for the likelihood of being prescribed a hypnotic and/or an anxiolytic when multimorbid		
	No of patients	Men	Women	No of patients	Men	Women	No of patients	Men	Women	Prevalence of Multimorbidity (%)	All patients	Men	Women
<1–19	182	72	110	312	167	145	354	150	204	42	6.7 (5.8–7.7)	5.8 (4.7–7.1)	7.7 (6.4–9.3)
20–29	238	84	154	779	337	442	1,731	573	1,158	63	6.5 (6.0–7.1)	6.5 (5.7–7.4)	6.3 (5.6–7.0)
30–39	222	93	129	875	388	487	2,974	999	1,975	73	7.1 (6.6–7.6)	6.8 (6.0–7.6)	6.8 (6.2–7.5)
40–49	140	51	89	687	288	399	4,054	1,287	2,767	83	7.7 (7.1–8.3)	7.2 (6.3–8.1)	7.3 (6.6–8.1)
50–59	126	52	74	518	225	293	5,510	1,817	3,693	89	9.4 (8.6–10.2)	8.0 (7.0–9.1)	9.7 (8.6–10.9)
60–69	66	26	40	302	130	172	5,014	1,782	3,232	93	13.4 (12.0–15.0)	10.9 (9.2–12.9)	15.6 (13.5–18.1)
70–79	30	11	19	135	59	76	3,783	1,318	2,465	96	20.0 (17.0–23.6)	15.0 (11.7–19.2)	25.1 (20.3–31.2)
80+	14	7	7	85	26	59	2,795	980	1,815	97	45.6 (37.2–56.0)	41.2 (28.9–58.7)	51.2 (39.7–66.0)
Total	1,018	396	622	3,693	1,620	2,073	26,215	8,906	17,309	85	14.9 (14.4–15.4)	12.7 (12.0–13.3)	16.0 (15.4–16.7)

All patients using a hypnotic, an anxiolytic or both either with no chronic condition, one chronic condition, or categorised as being multimorbid, with the odds ratios and 95 % confidence intervals for the likelihood of being prescribed a hypnotic and/or an anxiolytic when multimorbid

In this four-year period the total number of anxiolytics prescribed was 1.92 million defined daily doses (DDDs) [34], thereof 93 % benzodiazepines. During the same time 5.87 million DDDs of hypnotics were prescribed, 11 % benzodiazepines and nearly 88 % Z-drugs, hence in the combined group of hypnotics/anxiolytics, drugs with benzodiazepine structure were 31 % and Z-drugs 66 %.

When the ratio of multimorbid patients prescribed hypnotics/anxiolytics to the multimorbid patients in general is calculated it increases steadily as the number of chronic diseases within one patient increases (Table 7).

**Table 7:** Ratio of multimorbid patients prescribed hypnotics/anxiolytics to all multimorbid patients according to level of multimorbidity

No. of conditions	No. of multimorbid patients prescribed hypnotics/anxiolytics	All multimorbid patients	Ratio
2+	26,215	78,160	0.33
3+	21,573	50,554	0.43
4+	16,471	32,165	0.51
5+	11,581	19,664	0.59
6+	7,608	11,714	0.65
7+	4,657	6,593	0.71

The number of chronic conditions in the group of patients prescribed hypnotics/anxiolytics and the total population of multimorbid patients as well as the ratio between the groups

Of the 143,662 patients in the PHCCA cohort who did not have multimorbidity, 4711 were prescribed a hypnotic and/or an anxiolytic. On the other hand, 26,215 patients out of 78,160 multimorbid patients were prescribed these drugs. Thus, patients with multimorbidity were much more likely to be prescribed a hypnotic and/or an anxiolytic compared with patients without multimorbidity, OR = 14.9 (95 % CI = 14.4–15.4). Looking at men and women separately, women were more likely than men to be prescribed these drugs, OR = 16.0 (95 % CI = 15.4–16.7) for women versus OR = 12.7 (95 % CI = 12.0–13.3) for men, increasing with age, as shown in Fig. 4.

The mean age of patients who contacted the primary healthcare centres of the capital area was 36.4 years (men 35.7, women 37.0) compared with mean age of 36.8 years (men 36.1, women 37.4) for the total Icelandic population.

## Discussion

### Main findings

In our comprehensive health services study covering contacts within the primary healthcare in the capital area of Iceland during a four-year period we found the prevalence of multimorbidity to be 35 %. The population behind these contacts corresponds to approximately two-thirds of the total Icelandic population. Both prevalence and the incidence of hypnotic/anxiolytic prescriptions are high. More than 80 % of those aged 40 years or older prescribed hypnotics and/or anxiolytics are considered as being multimorbid, and 79 % of all incident patients are multimorbid. Around 83 % of the incident patients prescribed hypnotics/anxiolytics twice or more per year were multimorbid. Increased use of these drugs can at least partly, although not solely be explained by the extent of multimorbidity. The use of benzodiazepine anxiolytics (N05BA) and benzodiazepine hypnotics (N05CD) in Iceland is similar to their usage in other Nordic countries, but when comparing the use of the benzodiazepine related drugs, i.e. Z-drugs (N05CF), it is 1.7 to 3.3 times higher in Iceland than in the other countries [36]. In our study we found the incidence rate of hypnotic/anxiolytic prescriptions in Iceland, i.e. 19.4 cases per 1000 inhabitants/year, to be similar to what has been reported in a Norwegian study [32].

The prevalence of multimorbidity in our study is similar to what has been reported elsewhere although there are wide variations depending on the setting and the age of the population being studied [1–3, 5, 8]. As can be expected the prevalence increased with age. However, a decrease was noticed in the oldest age group. This could possibly in part be explained by the “survival of the fittest”, i.e. those with the greatest morbidity did not live long enough to reach this age. Also, a considerable number of the oldest patients with the greatest burden of frailty might have been cared for by the geriatric services without contacting the primary healthcare.

### Strengths and limitations

The group of patients contacting the healthcare centres during the four-year study period consisted of two-thirds of the total population with approximately the same mean age. It can be considered representative of the population as a whole and this broad coverage strengthens the generalisability of the study.

Almost all contacts within the primary care setting are registered in a computerised medical record kept in the same database as all issued prescriptions, most of them sent electronically to the pharmacy. In this database diagnoses are linked with the prescribed drugs through the unique personal identifier (ID). This can be regarded as strength. A previous Icelandic study found that the use of antidepressants, anxiolytics and hypnotics was most common among the socio-economically disadvantaged [37]. It may be considered a limitation that some important variables such as smoking, socioeconomic status and education are not systematically registered in our medical records, as often is the case in questionnaire based surveys. This has to be taken into account as we are not able to adjust for some of these confounders in our analysis. On the other hand, the medical records are more reliable regarding diagnoses and drug prescriptions in comparison to questionnaire based studies which can be regarded as strength.

The choice of chronic conditions in our study (Table 1) can be discussed. Most of them are distinct disease entities but others grouped together as chapter based conditions, e.g. cancer, mental health problems etc. This could possibly lead to underestimation of the prevalence of multimorbidity. On the other hand, the number of entities is large and can be considered as strength, as it has been shown that data for a minimum of twelve relevant chronic diseases are needed in order to obtain a fair evaluation of multimorbidity [3].

According to regulation in Iceland doctors are only allowed to prescribe a month’s supply of benzodiazepines and related drugs (Z-drugs). As a result, issuing only one prescription for the patient amounts to following clinical guidelines. Thus the number of prescriptions per patient per year indicates whether they are one time, intermittent or regular users. This strengthens the conclusions that can be drawn from this material.

We do not have figures on non-adherence in this study so prescriptions issued are the closest we come to actual use. The rate of primary non-adherence in Iceland was shown to be 6 % although not measured for hypnotics/anxiolytics [35]. This can be regarded a limitation.

In Iceland a GP-specialist referral system is not mandatory as a requirement by the health authorities, so instead of seeking medical attention at their general practitioner’s surgery patients may go directly to a specialist without a referral from the GP. Thus they would not be registered in the primary care material used in our study. This may be considered as a limitation.

### Implications

It is well known that multimorbidity often leads to polypharmacy. Most clinical guidelines refer to one specific index disease, sometimes with comorbidities, but without multimorbidity being used as a frame of reference. If these guidelines are followed strictly in a multimorbid patient one can end up with several drug related problems, e.g. interactions [38]. Hypnotics/anxiolytics with their numerous untoward effects may enhance the seriousness of the problem. Mortality rises as patients become older, frailer and more multimorbid, and untoward side effects and interactions of drugs could have an additive effect. It has been shown that the use of psychotropic drugs, hypnotics/anxiolytics included, and multimorbidity, jointly increased the risk of falls in the elderly [39]. Furthermore, it was found that a considerable number of patients with pain related diseases as fibromyalgia had both multiple chronic conditions and high use of sleep aids and benzodiazepines [40].

Studies on the side effects of drugs and drug related mortality are usually done in selected homogenous groups. However, our study confirms, that most of the people prescribed hypnotics/anxiolytics comprise a heterogeneous group with multiple chronic conditions which increase the risk of mortality, thereby limiting the possibility to explore the potential effect of hypnotics/anxiolytics on mortality separately without confounding factors influencing the result. Furthermore, it has been shown that more benzodiazepine prescriptions were issued for patients approaching death than for a control cohort, indicating that the use of these drugs was rather a consequence of disease discomfort, than death being a result of the use of these drugs [21].

Regardless of whether benzodiazepines and Z-drugs have a direct effect on mortality or not, the sum of all their deleterious effects may play a role in increased mortality and overshadow their beneficial effects. It should therefore be a priority to reduce the overprescribing of these drugs and to encourage clinicians to design, implement and disseminate guidelines with multimorbidity as a frame of reference in order to allow them and their patients with multimorbidity to make informed decisions about drug selection.

## Conclusions

This study reveals that in Iceland, as in many other countries, both the prevalence and the incidence of hypnotic/anxiolytic prescriptions are high. A considerable number of incident patients were prescribed benzodiazepines and Z-drugs for several times the recommended duration of therapy which could develop into long-term drug taking by these patients for an extended period, inconsistent with accepted medical practice. Solely explaining this by linear thinking, i.e. that “insomnia” leads to prescription of these drugs is probably simplistic, as the majority of patients prescribed these drugs are multimorbid having several chronic conditions, mainly mental and pain related diseases, which could lead to sleeping problems. There is a variety of concern with long-term use of these drugs as mentioned earlier in this paper, and their long-term efficacy has not been clearly established with some doubt having been cast on their short-term efficacy as well. Doctors should be urged to focus their attention to patients’ problems in the context of their multimorbidity and to be cautious when prescribing hypnotics for patients with multiple chronic conditions, bearing in mind that a holistic person focused approach is needed [4, 8, 41, 42]. They should be encouraged to discuss the duration of therapy with their patients before initiating benzodiazepine therapy in order to enable informed decisions about when these drugs should be discontinued. Multimorbidity per se is not directly an indication for prescribing hypnotics, and sleeping problems caused by the disease discomfort might in many instances be solved with non-pharmacological methods. It would be wise for family physicians to acquire skills in these forms of treatment.

## Abbreviations

ATC, anatomical therapeutic chemical classification; CI, confidence interval; CIRS, cumulative illness rating scale; DDD, defined daily dose; GP, general practitioner; ICD-10, international classification of diseases, 10^th^ edition; ICPC-2, international classification of primary care, version 2; ID, unique personal identifier; OR, odds ratio; PHCCA, primary health care of the capital area; Z-drugs, Benzodiazepine-related drugs. ATC-classification: N05CF
